# Genetic drivers of human plasma metabolites that determine mortality in heart failure patients with reduced ejection fraction

**DOI:** 10.3389/fcvm.2024.1409340

**Published:** 2024-07-09

**Authors:** Vandana Revathi Venkateswaran, Ruicong She, Hongsheng Gui, Jasmine A. Luzum, Timothy D. Bryson, Zack E. Malouf, L. Keoki Williams, Hani N. Sabbah, Stephen J. Gardell, David E. Lanfear

**Affiliations:** ^1^ Center for Individualized and Genomic Medicine Research, Henry Ford Hospital, Detroit, MI, United States; ^2^ Department of Public Health Science, Henry Ford Health, Detroit, MI, United States; ^3^ Department of Clinical Pharmacy, University of Michigan College of Pharmacy, Ann Arbor, MI, United States; ^4^ Cardiovascular Division, Department of Medicine, Henry Ford Hospital, Detroit, MI, United States; ^5^ Translational Research Institute, Advent Health, Orlando, FL, United States

**Keywords:** plasma metabolites, heart failure, GWAS, QTL, mediation analysis

## Abstract

**Background:**

Heart failure with reduced ejection fraction (HFrEF) remains a significant public health issue, with the disease advancing despite neurohormonal antagonism. Energetic dysfunction is a likely contributor to residual disease progression, and we have previously reported a strong association of plasma metabolite profiles with survival among patients with HFrEF. However, the genetic and biologic mechanisms that underlie the metabolite-survival association in HFrEF were uncertain.

**Methods and results:**

We performed genetic mapping of the key metabolite parameters, followed by mediation analyses of metabolites and genotypes on survival, and genetic pathway analyses. Patients with HFrEF (*n* = 1,003) in the Henry Ford Pharmacogenomic Registry (HFPGR; 500 self-reported Black/African race patients [AA], 503 self-reported White/European race patients [EA], and 249 deaths over a median of 2.7 years) with genome-wide genotyping and targeted metabolomic profiling of plasma were included. We tested genome-wide association (GWA) of single nucleotide polymorphisms (SNPs) with the prognostic metabolite profile (PMP) and its components; first stratified by race, and then combined via meta-analysis for the entire cohort. Seven independent loci were identified as GWA significant hits in AA patients (3 for PMP and 4 for individual metabolites), one of which was also significant in the entire cohort (rs944469). No genome wide significant hits were found in White/EA patients. Among these SNPs, only rs35792152, (a hit for 3.HBA) tended to be associated with mortality in standard survival analysis (HR = 1.436, *p* = 0.052). The mediation analyses indicated several significant associations between SNPs, metabolites, and mortality in AA patients. Functional annotation mapping (FUMA) implicated inflammation, DNA metabolic, and mRNA splicing processes.

**Conclusions:**

GWAS of key metabolites and survival along with FUMA pathway analysis revealed new candidate genes which unveiled molecular pathways that contribute to HF disease progression via metabolic and energetic abnormalities.

## Introduction

Heart failure (HF) is one of the most common, costly, and deadly diseases. According to the American Heart Association, there are almost 900,000 new cases annually in the U.S., an estimated prevalence of 5.7 million (1). HF remains high risk even in the setting of optimal guideline directed medical therapy (2, 3). Therefore, a deeper understanding of the residual HF disease burden and associated pathophysiology toward novel interventions is critically needed. Energetics, mitochondrial function, and substrate utilization are perturbed in HFrEF and likely contribute to disease progression (4–6). However, the pathways that remain incompletely understood and are not entirely related to the well-established neurohormonal abnormalities (7). Certain circulating metabolites are associated with HF disease progression and outcomes (8–11). Similarly, a validated multi-metabolite profile (13 metabolites, HR = 1.76) is an independent predictor of mortality in HFrEF patients. This supports the notion of a key role for energetic abnormalities in HFrEF and identifies these circulating metabolites as potentially valuable surrogate markers.

While genome-wide association studies (GWAS) have illuminated disease mechanisms in many conditions, GWAS on clinical HF phenotype alone requires very large sample sizes and lacks a mechanistic focus, limiting downstream interpretation. On the other hand, GWAS power may be enhanced when focused on important mechanistic surrogates for the disease, sometimes termed “endophenotypes”. For example, this approach has been successful in asthma (12) identifying novel causative pathways for hay fever (13), and in Alzheimer's disease where it allowed identification of novel variants with increased Alzheimer's Disease (14). In the case of HF, there have been a few candidate genes discovered in large GWAS studies (15), and yet, this approach may not be sensitive. Relevant to this, metabolomics has proven to be an advantageous approach in obtaining unique insights about underlying disease states (16–21). Data from both ours and other studies show that the plasma metabolite profile is associated with HF prognosis (21, 22). Hence, plasma metabolite levels in HF patients could be an attractive endophenotype to map in order to better understand the genetic and mechanistic underpinnings of HF progression.

In this study, we tested genetic association of prognostic metabolites and assessed mediation between the genetic factors and metabolites with survival outcomes. We then performed pathway analysis to identify key processes and groups of related genes that may be novel therapeutic targets in HF.

## Methods

### Sample collection

Participants for this study were enrolled into the Henry Ford heart failure Pharmacogenomic registry (HFPGR), the details of which are previously described (22). The study was approved by the Institutional Review Board at Henry Ford Health System in Detroit, MI, USA. After being educated about the study all participants provided written informed consent at the time of enrollment. Briefly, the registry consists of >1,700 HF patients in total that met the Framingham criteria for HF and who were covered for at least 1 year by the affiliated health insurance plan (the “Health Alliance Plan”) and primarily received their care through Henry Ford Health System. All patients have detailed clinical and electronic health record data, claims collected as part of the registry, and had donated blood for DNA and biomarker analyses. At the time of enrollment, fasting blood samples were collected and centrifuged to isolate plasma, aliquoted and stored at −70 °C until being assayed. This study is focused on HFrEF patients defined here as having at least one an ejection fraction (EF) <50% measured by any modality. From this registry, we collected patients with reduced EF, and they were separated into two groups according to their self-reported race: White (EA) and African American (AA). After quality control (QC), i.e., accounting for missing SNP and metabolite data, there were a total of 1,003 participants (500 AA and 503 EA) for this study.

### DNA genotyping

Genotyping was performed on all enrolled participants using the Axiom Biobank array (Affymetrix Inc, Santa Clara, CA). This array contained ∼600k SNPs including: (1) 300k GWAS markers with minor allele frequencies >1%, (2) >250k with low frequency (<1%) coding variants from the exome sequencing project, and (3) additional 50k variants to improve African race coverage (YRI Booster). This array provides excellent coverage of genomic variation, capturing 90% in European race and 80% in African race patients; and it also allows for ancestral quantification and admixture mapping. Standard quality control steps were adopted to clean the raw genotypes from the array (23). Clean genotypes were submitted to Michigan Imputation Server for imputation to 1,000 Genome Reference panel Phase 3 v5 (24). To avoid population differences, imputations were done separately for EA and AA. Only high-quality genotyped and imputed SNPs were included in the downstream association analysis. Minor allele frequency (MAF) cut-off was set at 0.05, and imputation score (r^2^) cut-off was set at 0.5.

### Metabolomic profiling

Quantitative and targeted metabolite profiling of individual amino acids (AA), organic acids (OA), and acylcarnitines (AC) were performed at the Sanford Burnham Prebys Medical Discovery Institute using HPLC/mass spectrometry or GC/mass spectrometry (22). Targeted metabolite profiling of 8 OAs, 23 AAs, and 57 ACs were measured using plasma samples (see Supplementary Material). A prognostic metabolite profile (PMP) that significantly predicted HF mortality was derived and validated in our cohort (22). This PMP was the primary independent variable for the metabolite quantitative trait loci (mQTL) GWAS. As secondary GWAS analyses, we analyzed the 13 individual metabolites that had significant independent association to mortality and were included in the PMP [Arginine, Leucine, Phenylalanine, Valine, C2, C4.Isobutyryl, C5.Isovaleryl, C5.DC, C18.1, 3-hydroxy butyric acid (3HBA), Succinate, Fumarate, and α-ketoglutarate (KG)]. We used these 14 molecular phenotypes (i.e., the PMP and each of the 13 individual metabolites) to search for mQTL associations in this study.

### Statistical analyses

To perform genome-wide association analysis on the metabolites that are potential endotypes of heart failure survival, typical GWAS protocol was used to link genome-wide SNPs with each metabolite and overall PMP. SNPs were coded as 0, 1 or 2 copies of the minor alleles. The primary analysis was performed for PMP and then secondary analyses for each of its component metabolites. A linear regression model implemented in PLINK was tested between SNP dosage and quantitative traits, with adjustment of the top 5 principal components (PC) (25). PCs were estimated from linkage disequilibrium pruned SNP sets using R package “Genesis” (26). Single variant association was done separately for EA and AA. To adopt a more powerful two-stage GWAS analysis across ethnic groups, AA GWAS was used as discovery analysis, while EA GWAS was used for replication. METAL was then used to combine their summary statistics together by fixed-effect meta-analysis (27). The significance threshold was set at 5 × 10^−8^ for SNP-level analysis.

### Bioinformatics functional annotation and pathway analyses

SNPs reaching genome-wide significance were interrogated for functional annotation (gene disrupting, expression quantitative trait locus, genomic regulation) that may link to HF pathology by public resources including The Encyclopedia of DNA Elements (ENCODE), Genotype-Tissue Expression (GTEx) and Human Metabolome database (HMDB) (28, 29) and metabolomics GWAS server (http://metabolomics.helmholtz-muenchen.de/gwas/).

Gene and gene-set association analyses were performed to identify functional pathways enriched by significant SNPs or genes. ENCODE annotation was used to mark functional genomic regions/genes. Combining the evidence of association from all SNPs within each gene/region was done using an extended Simes procedure to yield an overall gene-based association *p*-value (30). The gene-focused approach enabled assessment of pathways to allow investigation of multi-site effects and leverage other pre-existing data such as known protein-protein interactions or regulatory pathways (i.e., FUMA).

### Mediation analysis and heart failure mortality

Two lists of SNPs were included in the mediation analysis, which was used to build up the causal relationship between candidate SNPs, metabolites, and HF mortality. The first list was from mQTL analysis in this study, and the second list was from another independent GWAS on HF survival phenotype. Metabolites were treated as mediators, and HF mortality was treated as the outcome. Both model-based mediation analysis and its corresponding sensitivity analysis were done by the R package “Mediation” (31). A significant mediation effect was defined when mediation analysis returned a *p*-value < 0.01 and sensitivity analysis returned a *p*-value >0.05. The top associated SNPs, genes, or pathways were tested for mortality association in Cox proportional models adjusted for the first 5 PCs, clinical risk score (MAGGIC score) (32) and NT-proBNP levels. Multiple testing was corrected by Bonferroni method using the total number of genetic risk variables tested (i.e., the total number of hits from mQTL analysis).

## Results

The baseline characteristics for the HFrEF patients in our analytic cohort are shown in Table 1. Among a total of 1,003 patients, 35% were female, 49% self-identified as AA, and the cohort average age was 68 years. The average ejection fraction was 34.9%, and 57% of the cohort had HF of ischemic origin.

**Table 1:** Baseline characteristics of HFrEF patients in HFPGR analytic cohort (*n* = 1,003).

Characteristic	Value
Female, *N*	353 (35%)
Age, years	68 (±12)
African American, *N*	500 (51%)
Systolic blood pressure, mmHg	129 ± 23
Heart rate, bpm	71 (±13)
Ischemic, *N*	568 (57%)
COPD, *N*	222 (22%)
A-Fib, *N*	279 (28%)
Stroke/TIA, *N*	90 (8.97%)
Diabetes, *N*	412 (41%)
LVEF, %	34.9 (±11)
NTproBNP, pg/ml	354.02 (±378.35)
Creatinine, mg/dl	1.30 (±0.99)
MAGGIC score	19.22 (±7.78)
KCCQ overall	82 (±21)
Follow up time, days	995 (591)
Death	249 (24.8%)
NYHA Class I	582 (58.03%)
NYHA Class II	182 (18.15%)
NYHA Class III	120 (11.96%)
NYHA Class IV	119 (11.86%)
ACE use	482 (48.06%)
ARB use	196 (19.54%)
Beta blocker use	692 (68.99%)

**Figure 1:**
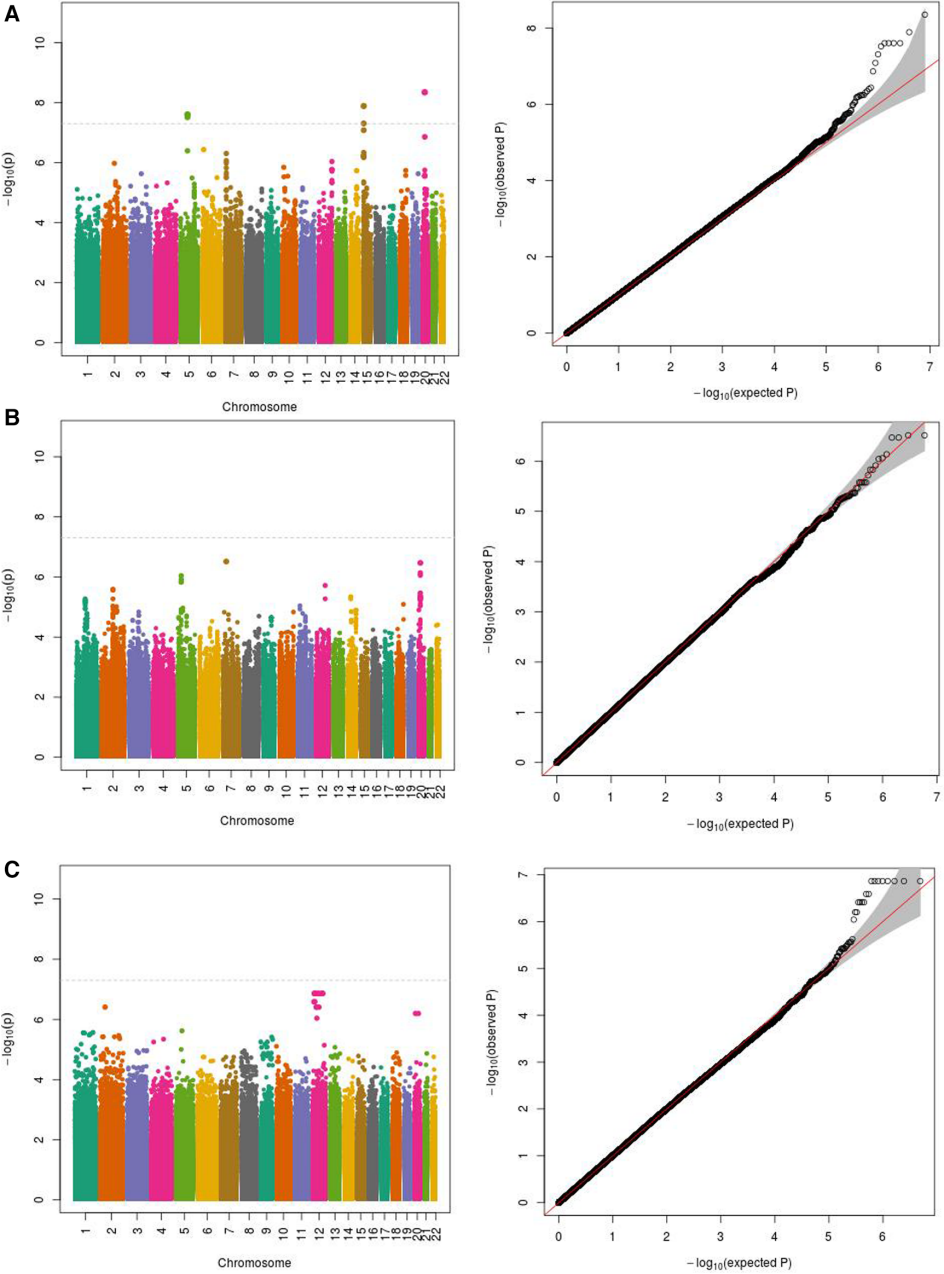
Manhattan and QQ plots for PMP association in (**A**) African Americans (−500 subjects); (**B**) European Americans; (**C**) meta-analysis. (**A**) PMP as phenotype in African Americans (500 subjects). (**B**) PMP as phenotype in European Americans (503 subjects). (**C**) PMP meta-analytic GWAS in African American and European Americans subjects (*n* = 1,003).

**Figure 2:**
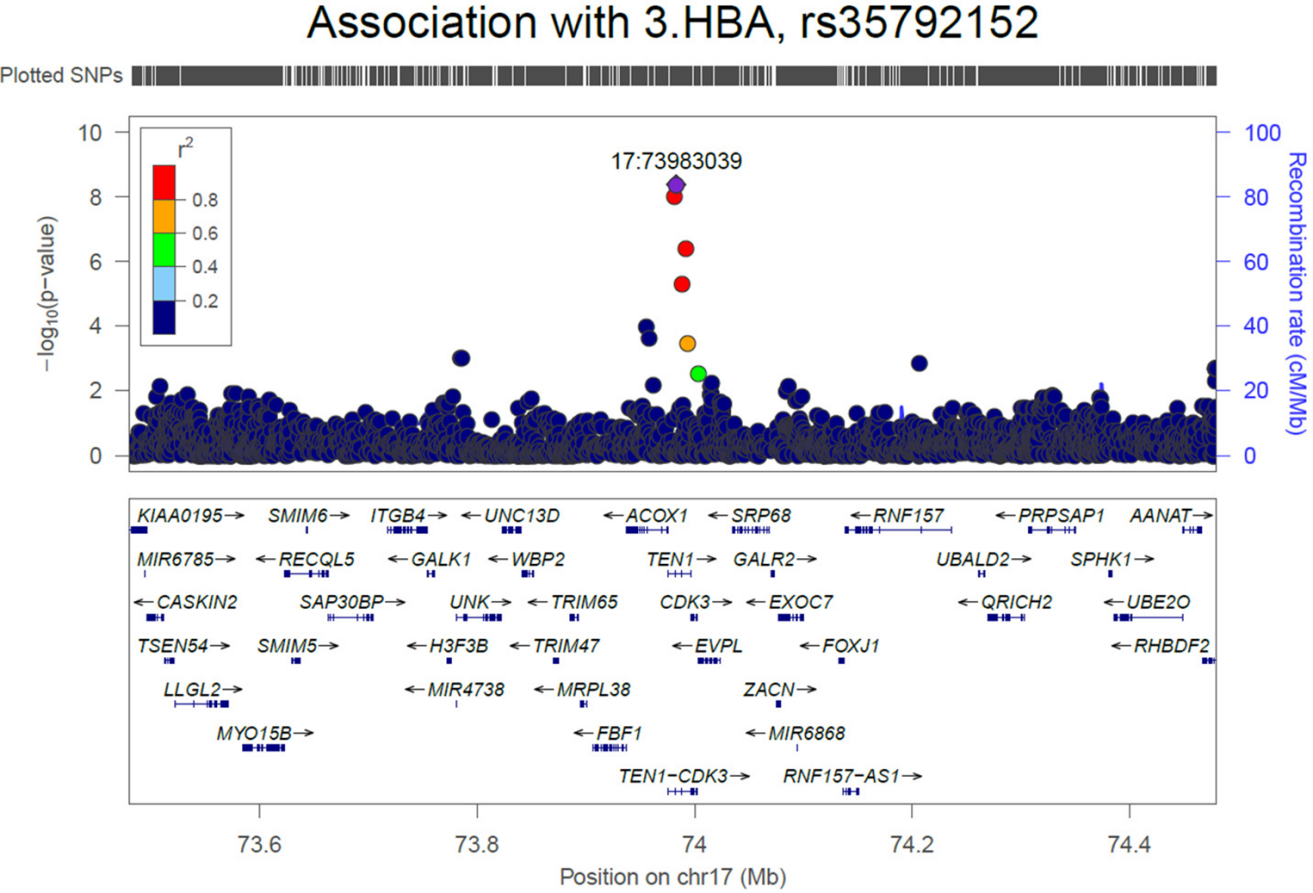
Regional plot for association of SNPs with 3.HBA in African American population.

We initially performed systematic genetic analysis for the multi-metabolite profile metabolite levels (PMP) in HFrEF patients, these results are depicted in Figure 1. Among AA participants, 3 SNPs were identified that met whole-genome significance (*p* < 5 × 10^−8^), while no SNPs were significant among EA participants (Table 2). Among the 3 significant SNPs, one was located in a protein coding gene (ARHGEF28) and the other two were in inter-genic regions. We then examined the individual metabolites that make up the previously published prognostic profile. An additional 4 independent SNPs were identified as GWAS hits for human metabolites in AA HFrEF patients. The multi-race meta-analytic GWAS resulted in no SNPs meeting genome wide significance. No significant SNPs were identified in the EA patients. Among AA patients, one metabolite hit, rs35792152 for 3-HBA (Figure 2), was moderately associated with HF mortality (HR = 1.436, *p* = 0.052).

**Table 2:** Top associated SNPs with metabolite level.

Chr	SNP	MAF	Info score	Gene	Pop	Metabolite	Beta	*P*
1	rs4402119	0.193	0.91	Intergenic	AA	Arginine	0.442	1.35 × 10^−8^
17	rs35792152	0.064	0.83	TEN1, Near ACOX1 (upstream)	AA	3.HBA	−0.803	4.08 × 10^−9^
19	rs4643456	0.172	0.85	LOC284395 (intron)	AA	C4 Isobutyryl	0.51	5.97 × 10^−9^
8	rs73281346	0.132	0.72	Intergenic	AA	Fumarate	0.526	4.03 × 10^−8^
5	rs6453017	0.461	0.76	Near ARHGEF28 (upstream)	AA	PMP	0.181	2.51 × 10^−8^
15	rs11638945	0.145	0.84	Intergenic	AA	PMP	0.269	1.29 × 10^−8^
20	rs944469	0.095	0.9	Intergenic	AA	PMP	0.321	4.49 × 10^−9^

To gain a composite view of all genetic associations and use this to identify key pathways, we took a systems biology approach using FUMA, leveraging the primary GWAS output. Top associated gene-sets using FUMA for the multi-metabolite profile are shown in Table 3. Results derived using FUMA highlighted some additional intriguing gene ontology groups such as endoribonuclease activity, regulation of RNA splicing, Holliday junction resolving, and defense from virus. Finally, mediation models were used to assess the relationship of genetic signals, metabolite traits and clinical outcome (mortality), results of which are summarized in Table 4. Mediation analysis of individual SNP hits did not reveal any metabolites mediating the effect of single SNP genotypes on HF mortality (all *p* > 0.05).

**Table 3:** Top associated gene sets from FUMA analysis.

Group	Pathway name	No. of genes	*P*-value	FDR
Meta	Endodeoxyribonuclease_activity_producing_3_phosphomonoesters	10	0.000002485	0.0222609
Meta	Negative_regulation_of_mrna_splicing_via_spliceosome	18	0.000002876	0.0222609
Meta	Negative_regulation_of_rna_splicing	23	0.000011771	0.0522135
Meta	Endodeoxyribonuclease_activity	37	0.000013491	0.0522135
Meta	Holliday_junction_resolvase_complex	5	0.000031466	0.0819332
Meta	Regulation_of_mrna_splicing_via_spliceosome	89	0.000031755	0.0819332
Meta	Crossover_junction_endodeoxyribonuclease_activity	7	0.000038907	0.0851416
Meta	Maternal_process_involved_in_parturition	7	0.000043998	0.0851416
AA	Defense_response_to_virus	224	0.000002676	0.0414318

**Table 4:** Mediation analysis between SNP, metabolite, and mortality.

Genetic variants	Population	Model 1 (Survival – Metabolite)			Model 2 (Metabolite – SNP)		Model 3 (Survival – SNP)		Model 4 (mediation)	
		Metabolite	beta1	*P1*	beta2	*P*2	beta3	*P3*	% Mediated	*P*4
rs6453017_C	African American (AA)	PMP	0.420	**0.0214**	0.181	**2.51 × 10^−8^**	−0.137	1.68 × 10^−1^	139	0.35
rs11638945_A					0.269	**1.29 × 10^−8^**	0.202	1.10 × 10^−1^	57.4	**<2e-16**
rs944469_G					0.321	**4.49 × 10^−9^**	−0.147	3.67 × 10^−1^	110	0.058
rs35792152_C		3HBA	−0.043	0.516	−0.803	**4.08 × 10^−9^**	0.478	**3.32 × 10^−3^**	4.09	0.73
rs6453017_C	European American (EA)	PMP	1.122	**7.93 × 10^−8^**	−0.006	8.45 × 10^−1^	−0.135	1.33 × 10^−1^	23	0.67
rs11638945_A					−0.022	4.80 × 10^−1^	−0.044	6.32 × 10^−1^	28.5	0.55
rs35792152_C		3HBA	−0.251	**0.000291**	0.095	5.22 × 10^−1^	0.114	5.89 × 10^−1^	−1.77	0.86
rs6453017_C	Meta analysis (All samples)	PMP	0.681	**4.83 × 10^−7^**	0.084	**1.83 × 10^−4^**	−0.151	**2.17 × 10^−2^**	118	0.62
rs11638945_A					0.068	**9.17 × 10^−3^**	0.021	7.71 × 10^−1^	73.5	0.228
rs944469_G					0.321	**4.49 × 10^−9^**	−0.147	3.67 × 10^−1^	NA	NA
rs35792152_C		3HBA	−0.137	**0.00439**	−0.419	**3.02 × 10^−5^**	0.293	**2.22 × 10^−2^**	12.5	**0.032**

The values in bold highlight the significant *P*-values.

## Discussion

Metabolomic profile is a potentially useful endophenotype for HF progression since plasma metabolite levels are associated with risk of death in HF, and are linked to mitochondrial function and energetics, which represents a key pathophysiologic pathway in HF. Thus, the study of genetic influences on metabolites as endophenotypes may provide a short-cut to understanding the mechanisms involved that contribute to worsening HFrEF. The present study examined this, utilizing a combined approach by predicting mortality using genetic GWAS hits combined with a metabolite profile to identify novel candidate genes for HF (COX1, ARHGEF28, and TEN1) and underscoring key gene ontology groups (33) that likely have impact in HF development and progression (metabolic and apoptotic pathways, among others).

We identified several genome wide significant hits for the PMP, but interestingly, all of them appear in the AA-only analysis. The reason for this is unclear. While the lack of findings across both cohorts could reflect chance findings, it also could be that the greater genetic diversity present in AA patients provides greater power in this moderate sized study. One SNP was near (approximately 3 kb upstream) a known coding gene, Rho Guanine Nucleotide Exchange Factor 28 (ARHGEF28). While this precise gene has not previously been linked to HF, GEFs are involved in processes across a wide range of tissues interacting with integrins and are linked to autophagy and apoptotic pathways; both processes known to be active in the setting of HF as well as being linked to mitochondrial and energetic cellular functions. Indeed, other GEF family members have been linked to cardiac remodeling and cardiac fibrosis in model systems (34, 35).

We identified only one SNP (rs35792152) that was significant for one of the prognostic metabolites (3HBA) and that was also associated with mortality. This SNP lies in or near multiple genes; it is within Telomere Length Regulation Protein gene (*TEN1*) and *TEN1-CDK3* (a read-through long non-coding RNA gene), and near (5 kb upstream) Acyl-CoA Oxidase 1 (*ACOX1*). TEN1 is a key part of a protein complex involved in telomere maintenance during DNA replication (36). Telomere length and maintenance is linked to aging and senescent cell processes, but also more specific links to energetic function and age-related diseases (like HF) have been widely proposed (37). More obviously, ACOX1 catalyzes the initial and rate-limiting step in peroxisomal beta-oxidation of fatty acids, a process with emerging implications for heart failure (38). There are intriguing links between TEN-CDK3, a lncRNA gene, with dextro-cardia and cardiomyopathy. In considering the possible gene links of this SNP, the fact that it is associated with 3HBA levels and is located near ACOXI could be seen as pointing to energetic mechanisms or substrate utilization. However, as described, the other potential candidates also have plausible biologic mechanisms. Further investigation is needed to clarify possible causation and mechanism.

One possible limitation of the study is that the plasma metabolite profile may not reflect HF disease progression *per se*. However, this is unlikely given that most patients with HFrEF die from cardiac complications and not another disease. Another possible limitation of the study is that the plasma metabolite profile may reflect HF progression but not myocardial energetics specifically. With regards to alternative approaches to the metabolomic studies themselves, one could consider global/untargeted metabolomics. However, existing literature on metabolomics in cardiovascular disease has focused our attention on several very promising metabolite classes including ACs, AAs and OAs, and the strength of our data associating these metabolites with survival in HF patients suggests we stay focused on fewer, quantifiable analytes. Moreover, a formidable challenge of global metabolomics is identification of unknown (unannotated) metabolites, which is key for elucidating mechanism and developing new intervention strategies. Future studies are needed to look over more metabolomics wide markers, not just targeted assays. Lastly, an additional limitation of the study is the relatively small sample size (*N* = 1,003).

Moreover, the patient metabolite profile was measured only at one time point i.e., the time of study enrollment. The metabolite profile was not taken in relation to any disease stage, and no direct replication of the profile was performed. We have developed a novel method to link genetic variants with changes in plasma metabolite levels and HF mortality. Several causal relationships were uniquely significant in the AA population, suggesting this technique could be useful in generating prognostic factors in at-risk populations.

## Data Availability

The datasets presented in this study can be found in online repositories. The names of the repository/repositories and accession number(s) can be found below: https://www.ncbi.nlm.nih.gov/gap/, phs001501.v1.p1.

## References

[1] Virani SS Alonso A Benjamin EJ Bittencourt MS Callaway CW Carson AP Heart disease and stroke statistics-2020 update: a report from the American heart association. Circulation. (2020) 141:e139–596. 10.1161/CIR.0000000000000757 31992061

[2] Greene SJ Khan MS. Quadruple medical therapy for heart failure: medications working together to provide the best care. J Am Coll Cardiol. (2021) 77:1408–11. 10.1016/j.jacc.2021.02.006 33736822

[3] Bauersachs J. Heart failure drug treatment: the fantastic four. Eur Heart J. (2021) 42:681–3. 10.1093/eurheartj/ehaa1012 33447845 PMC7878007

[4] Jarreta D Orús J Barrientos A Miró O Roig E Heras M Mitochondrial function in heart muscle from patients with idiopathic dilated cardiomyopathy. Cardiovasc Res. (2000) 45:860–5. 10.1016/S0008-6363(99)00388-0 10728411

[5] Lopez R Marzban B Gao X Lauinger E Van den Bergh F Whitesall SE Impaired myocardial energetics causes mechanical dysfunction in decompensated failing hearts. Function (Oxf). (2020) 1:zqaa018. 10.1093/function/zqaa018 33074265 PMC7552914

[6] Turer AT. Using metabolomics to assess myocardial metabolism and energetics in heart failure. J Mol Cell Cardiol. (2013) 55:12–8. 10.1016/j.yjmcc.2012.08.025 22982115

[7] Packer M. The neurohormonal hypothesis: a theory to explain the mechanism of disease progression in heart failure. J Am Coll Cardiol. (1992) 20:248–54. 10.1016/0735-1097(92)90167-L 1351488

[8] Hunter WG Kelly JP McGarrah RW 3rd Khouri MG Craig D Haynes C Metabolomic profiling identifies novel circulating biomarkers of mitochondrial dysfunction differentially elevated in heart failure with preserved versus reduced ejection fraction: evidence for shared metabolic impairments in clinical heart failure. J Am Heart Assoc. (2016) 5(8):e003190. 10.1161/JAHA.115.003190 27473038 PMC5015273

[9] Lopaschuk GD Karwi QG Tian R Wende AR Abel ED. Cardiac energy metabolism in heart failure. Circ Res. (2021) 128:1487–513. 10.1161/CIRCRESAHA.121.318241 33983836 PMC8136750

[10] Willner S Gardell S She R Li J Zeld N Williams KL Circulating metabolomic profile predicts change in ejection fraction in heart failure patients. J Card Fail. (2023) 29:569. 10.1016/j.cardfail.2022.10.061

[11] Tahir UA Katz DH Zhao T Ngo D Cruz DE Robbins JM Metabolomic profiles and heart failure risk in black adults: insights from the Jackson heart study. Circ Heart Fail. (2021) 14:e007275. 10.1161/CIRCHEARTFAILURE.120.007275 33464957 PMC9158510

[12] Henderson AJ. Childhood asthma phenotypes in the twenty-first century. Breathe. (2014) 10:100–8. 10.1183/20734735.014613

[13] Ferreira MA Matheson MC Tang CS Granell R Ang W Hui J Genome-wide association analysis identifies 11 risk variants associated with the asthma with hay fever phenotype. J Allergy Clin Immunol. (2014) 133:1564–71. 10.1016/j.jaci.2013.10.030 24388013 PMC4280183

[14] Harold D Abraham R Hollingworth P Sims R Gerrish A Hamshere ML Genome-wide association study identifies variants at CLU and PICALM associated with Alzheimer’s disease. Nat Genet. (2009) 41:1088–93. 10.1038/ng.440 19734902 PMC2845877

[15] van der Ende MY Said MA van Veldhuisen DJ Verweij N van der Harst P. Genome-wide studies of heart failure and endophenotypes: lessons learned and future directions. Cardiovasc Res. (2018) 114:1209–25. 10.1093/cvr/cvy083 29912321

[16] Shah SH Kraus WE Newgard CB. Metabolomic profiling for the identification of novel biomarkers and mechanisms related to common cardiovascular diseases: form and function. Circulation. (2012) 126:1110–20. 10.1161/CIRCULATIONAHA.111.060368 22927473 PMC4374548

[17] Shah SH Sun JL Stevens RD Bain JR Muehlbauer MJ Pieper KS Baseline metabolomic profiles predict cardiovascular events in patients at risk for coronary artery disease. Am Heart J. (2012) 163:844–50.e1. 10.1016/j.ahj.2012.02.005 22607863

[18] Salek R Cheng KK Griffin J. The study of mammalian metabolism through NMR-based metabolomics. Methods Enzymol. (2011) 500:337–51. 10.1016/B978-0-12-385118-5.00017-7 21943905

[19] Griffin JL Atherton H Shockcor J Atzori L. Metabolomics as a tool for cardiac research. Nat Rev Cardiol. (2011) 8:630–43. 10.1038/nrcardio.2011.138 21931361

[20] Dunn WB Broadhurst DI Deepak SM Buch MH McDowell G Spasic I Serum metabolomics reveals many novel metabolic markers of heart failure, including pseudouridine and 2-oxoglutarate. Metabolomics. (2007) 3:413–26. 10.1007/s11306-007-0063-5

[21] Ahmad T Kelly JP McGarrah RW Hellkamp AS Fiuzat M Testani JM Prognostic implications of long-chain acylcarnitines in heart failure and reversibility with mechanical circulatory support. J Am Coll Cardiol. (2016) 67:291–9. 10.1016/j.jacc.2015.10.079 26796394 PMC5429585

[22] Lanfear DE Gibbs JJ Li J She R Petucci C Culver JA Targeted metabolomic profiling of plasma and survival in heart failure patients. JACC Heart Fail. (2017) 5:823–32. 10.1016/j.jchf.2017.07.009 29096792 PMC5679305

[23] Weale ME. Quality control for genome-wide association studies. Methods Mol Biol. (2010) 628:341–72. 10.1007/978-1-60327-367-1_19 20238091

[24] Das S Forer L Schönherr S Sidore C Locke AE Kwong A Next-generation genotype imputation service and methods. Nat Genet. (2016) 48:1284–7. 10.1038/ng.3656 27571263 PMC5157836

[25] Purcell S Neale B Todd-Brown K Thomas L Ferreira MA Bender D PLINK: a tool set for whole-genome association and population-based linkage analyses. Am J Hum Genet. (2007) 81:559–75. 10.1086/519795 17701901 PMC1950838

[26] Conomos MP Miller MB Thornton TA. Robust inference of population structure for ancestry prediction and correction of stratification in the presence of relatedness. Genet Epidemiol. (2015) 39:276–93. 10.1002/gepi.21896 25810074 PMC4836868

[27] Willer CJ Li Y Abecasis GR. METAL: fast and efficient meta-analysis of genomewide association scans. Bioinformatics. (2010) 26:2190–1. 10.1093/bioinformatics/btq340 20616382 PMC2922887

[28] Consortium EP. An integrated encyclopedia of DNA elements in the human genome. Nature. (2012) 489:57–74. 10.1038/nature11247 22955616 PMC3439153

[29] Consortium GT. The genotype-tissue expression (GTEx) project. Nat Genet. (2013) 45:580–5. 10.1038/ng.2653 23715323 PMC4010069

[30] Li MX Gui HS Kwan JS Sham PC. GATES: a rapid and powerful gene-based association test using extended simes procedure. Am J Hum Genet. (2011) 88:283–93. 10.1016/j.ajhg.2011.01.019 21397060 PMC3059433

[31] Tingley DYT Hirose K Keele L Imai K. Mediation: r package for causal mediation analysis. J Stat Softw. (2014) 59:1–38. 10.18637/jss.v059.i0526917999

[32] Mafort Rohen F Xavier de Avila D Martins Cabrita Lemos C Santos R Ribeiro M Villacorta H. The MAGGIC risk score in the prediction of death or hospitalization in patients with heart failure: comparison with natriuretic peptides. Rev Port Cardiol. (2022):S0870-2551(22)00363-8. 10.1016/j.repc.2021.07.015 36202681

[33] Mahajan P Fiehn O Barupal D. IDSLGOA: gene ontology analysis for interpreting metabolomic datasets. Sci Rep. (2024) 14(1):1299. 10.1038/s41598-024-51992-x PMC10788336 38221536

[34] Takefuji M Kruger M Sivaraj KK Kaibuchi K Offermanns S Wettschureck N. RhoGEF12 controls cardiac remodeling by integrating G protein- and integrin-dependent signaling cascades. J Exp Med. (2013) 210:665–73. 10.1084/jem.20122126 23530122 PMC3620351

[35] Numaga-Tomita T Kitajima N Kuroda T Nishimura A Miyano K Yasuda S TRPC3-GEF-H1 Axis mediates pressure overload-induced cardiac fibrosis. Sci Rep. (2016) 6:39383. 10.1038/srep39383 27991560 PMC5171702

[36] Price CM Boltz KA Chaiken MF Stewart JA Beilstein MA Shippen DE. Evolution of CST function in telomere maintenance. Cell Cycle. (2010) 9:3157–65. 10.4161/cc.9.16.12547 20697207 PMC3041159

[37] Gao X Yu X Zhang C Wang Y Sun Y Sun H Telomeres and mitochondrial metabolism: implications for cellular senescence and age-related diseases. Stem Cell Rev Rep. (2022) 18:2315–27. 10.1007/s12015-022-10370-8 35460064 PMC9033418

[38] Colasante C Chen J Ahlemeyer B Baumgart-Vogt E. Peroxisomes in cardiomyocytes and the peroxisome/peroxisome proliferator-activated receptor-loop. Thromb Haemost. (2015) 113:452–63. 10.1160/TH14-06-0497 25608554

