# Supplementary material for: Genetic drivers of human plasma metabolites that determine mortality in heart failure patients with reduced ejection fraction
Source: Front Cardiovasc Med. 2024 Jul 9;11:1409340. doi: 10.3389/fcvm.2024.1409340 (PMC11263106; doi:10.3389/fcvm.2024.1409340)
Supplement: Supplementary file 1 [file Table1.docx]

**Genetic Drivers of Human Plasma Metabolites That Determine Mortality in Heart Failure Patients with Reduced Ejection Fraction**

Vandana Revathi Venkateswaran^1^, Ruicong She ^2^, Hongsheng Gui ^1^, Jasmine A. Luzum^1,3^, Timothy D. Bryson ^1^, Zack E. Malouf ^4^, L. Keoki Williams ^1^, Hani N. Sabbah ^4^, Stephen J. Gardell ^5^, David E. Lanfear ^1,4^

^1^Center for Individualized and Genomic Medicine Research, Henry Ford Hospital, Detroit, MI;

^2^Department of Public Health Science, Henry Ford Health, Detroit, MI;

^3^Department of Clinical Pharmacy, University of Michigan College of Pharmacy, Ann Arbor, MI;

^4^Cardiovascular Division, Department of Medicine, Henry Ford Hospital, Detroit, MI;

^5^Translational Research Institute, Advent Health, Orlando, FL.

**Supplementary Material:

Table S1:** List of metabolites (8 OAs, 23 AAs, and 57 ACs) on which targeted metabolite profiling was measured using plasma samples**:**

**Organic Acids**

| **Type** | **Metabolite** |
| --- | --- |
| OA | Lactate |
| OA | Pyruvate |
| OA | 3-HBA |
| OA | Succinate |
| OA | Fumarate |
| OA | Malate |
| OA | a-KG |
| OA | Citrate |

**Amino Acids**

| **Type** | **Metabolite** |
| --- | --- |
| AA | 1-MethylHistidine |
| AA | 3-MethylHistidine |
| AA | Alanine |
| AA | Arginine |
| AA | Asparagine |
| AA | Aspartate |
| AA | Citrulline |
| AA | Glutamate |
| AA | Glutamine |
| AA | Glycine |
| AA | Histidine |
| AA | Isoleucine |
| AA | Leucine |
| AA | Lysine |
| AA | Methionine |
| AA | Ornithine3 |
| AA | Phenylalanine |
| AA | Proline |
| AA | Serine |
| AA | Threonine |
| AA | Tryptophan |
| AA | Tyrosine |
| AA | Valine |

**(C) Acyl Carnitines**

| **Type** | **Metabolite** |
| --- | --- |
| AC | C2 |
| AC | C3-DC |
| AC | C3 |
| AC | C4 Butyryl |
| AC | C4 Isobutyryl |
| AC | C4:1 |
| AC | C4-DC Methylmalonyl |
| AC | C4-DC Succinyl |
| AC | C4-OH |
| AC | C5 2-Methylbutyryl |
| AC | C5 Isovaleryl |
| AC | C5 Valeryl |
| AC | C5:1 |
| AC | C5-DC |
| AC | C5-OH |
| AC | C6 |
| AC | C6-OH |
| AC | C8:1-OH |
| AC | C8 |
| AC | C8-OH |
| AC | C10 |
| AC | C10-OH |
| AC | C12:1 |
| AC | C12 |
| AC | C12-OH |
| AC | C14:1 |
| AC | C14:1-OH |
| AC | C14:2 |
| AC | C14:2-OH |
| AC | C14 |
| AC | C14-OH |
| AC | C16:1 |
| AC | C16:1-OH |
| AC | C16:2 |
| AC | C16:2-OH |
| AC | C16 |
| AC | C16-OH |
| AC | C18:1 |
| AC | C18:1-OH |
| AC | C18:2 |
| AC | C18:2-OH |
| AC | C18 |
| AC | C18-OH |
| AC | C20:1 |
| AC | C20:1-OH |
| AC | C20:2 |
| AC | C20:2-OH |
| AC | C20:3 |
| AC | C20:4 |
| AC | C20 |
| AC | C20-OH/C22:6-DC |
| AC | C22:1 |
| AC | C22:2 |
| AC | C22:3 |
| AC | C22:4 |
| AC | C22:5 |
| AC | C22 |
